# A distinct molecular mutational profile and its clinical impact in essential thrombocythemia and primary myelofibrosis patients

**DOI:** 10.1186/s12885-020-6700-3

**Published:** 2020-03-12

**Authors:** Uzma Zaidi, Gul Sufaida, Munazza Rashid, Bushra Kaleem, Sidra Maqsood, Samina Naz Mukry, Rifat Zubair Ahmed Khan, Saima Munzir, Munira Borhany, Tahir Sultan Shamsi

**Affiliations:** 1 Department of Clinical Hematology, National Institute of Blood Diseases & Bone Marrow Transplantation, Karachi, Pakistan; 2 Department of Molecular Medicine, National Institute of Blood Diseases & Bone Marrow Transplantation, Karachi, Pakistan; 3 Department of Clinical Research, National Institute of Blood Diseases & Bone Marrow Transplantation, Karachi, Pakistan

**Keywords:** BCR-ABL negative myeloproliferative neoplasm, Essential thrombocythemia, Primary myelofibrosis, Overall survival, Leukaemic free survival

## Abstract

**Background:**

Classical MPNs including ET and PMF have a chronic course and potential for leukaemic transformation. Timely diagnosis is obligatory to ensure appropriate management and positive outcomes. The aim of this study was to determine the mutational profile, clinical characteristics and outcome of ET and PMF patients in Pakistani population.

**Methods:**

This was a prospective observational study conducted between 2012 and 2017 at NIBD. Patients were diagnosed and risk stratified according to international recommendations. Response to treatment was assessed by IWG criteria.

**Results:**

Of the total 137 patients analysed, 75 were ET and 62 were PMF. *JAK2* positivity was seen in 51 cases (37.2%), *CALR* in 41 cases (29.9%), while triple-negative in 17 (12.4%) cases. None of the patients in the present study were MPL positive. Overall survival for patients with ET and PMF was 92.5 and 86.0% respectively and leukaemia free survival was 100 and 91.6% respectively, at a median follow-up of 12 months. Leukaemic transformation occurred in 6.5% of MF patients; among them, *JAK2* mutation was frequently found. Molecular mutations did not influence the OS in ET whereas in PMF, OS was shortest in the triple-negative PMF group as compared to the *JAK2* and *CALR* positive patient groups.

**Conclusion:**

This study shows a different spectrum of molecular mutations in ET and PMF patients in Pakistani population as compared to other Asian countries. Similarly, the risk of leukaemic transformation in ET and PMF is relatively lower in our population of patients. The factors responsible for these phenotypic and genotypic differences need to be analysed in large scale studies with longer follow-up of patients.

## Background

Primary Myelofibrosis and Essential thrombocythemia are classical Philadelphia-negative myeloproliferative neoplasms (MPNs), characterized by stem cell-derived clonal proliferation of one or more of myeloid lineage cells. The incidence of the classical MPNs reported worldwide is approximately 0.5–6/100,000 per year. It is considered a disease of the elderly with peak incidence occurring in the 5th to 6th decades of life [1, 2]. MPNs have the tendency to progress into myelofibrosis and transform into acute leukaemia after a certain period which may vary with each subtype of MPN [3].

The latest advancements in the molecular pathogenesis of classical MPN have revealed that each subtype of MPN carries a specific driver mutation including *JAK2, CALR and MPL* or somatic mutations in *TET2, ASXL1, IDH, IKZF1, EZH2, DNMT3A, TP53, SF3B1, SRSF2, U2AF1* or other mutations [4]. The most recent revision of the classification of MPN published by the World Health Organization (WHO) has incorporated the presence of *CALR* and *MPL* mutations in the diagnostic criteria of PMF and ET based on the current evidences [5]. *CALR* mutations which are typically insertions or deletions and involve exon 9 have been reported in 60–90% of PMF and ET patients with unmutated *JAK2* or *MPL* [6]. The most frequent subtypes of *CALR* are Type-1 (L367fs*46) and Type-2 (K385FS*47) [7]. It is generally believed that driver mutations are crucial for the MPN phenotype whereas the other mutations are associated with disease progression and leukaemic transformation [8].

The clinical presentation of ET is heterogeneous ranging from asymptomatic thrombocytosis to life threatening bleeding or thrombosis involving the major vessels of the body [9]. Patients who present with extreme thrombocytosis (> 1500 × 10^9^/L) require vigilant monitoring because of the increased risk of haemorrhage due to acquired von Willebrand syndrome [10]. The risk of leukaemic transformation or progression into post-ET myelofibrosis increases with thrombosis, leucocytosis and increasing age [11]. On the other hand, typical clinical features of PMF include progressive anaemia, symptomatic splenomegaly, and various constitutional symptoms requiring treatment [12]. PMF is associated with a poor outcome and reduced life expectancy, with median survival durations ranging from 3.5 to 6 years, according to the previous studies [13]. Transformation into acute leukaemia occurs in approximately 20% of patients [14].

The diagnosis and management of MPNs in developing countries have always been challenging due to limited health resources. The molecular diagnostic facilities are limited to a few large tertiary care centres where access of patients from remote areas is difficult. Lack of awareness and delay in diagnosis results in suboptimal treatment, making the prognosis dismal in this part of the world.

In Pakistan, there is no well-defined cancer registry for MPN or other cancers, therefore data regarding the incidence, clinical presentation and outcome of patients suffering from different subtypes of MPN are scarce. Until 2012, molecular diagnostic facilities in our country were limited to PCR for *BCR-ABL* and *JAK2* mutations. This is the first study from Pakistan which includes the molecular diagnosis of MPN based on cytogenetic analysis, PCR for *JAK2, CALR* and *MPL* mutations. The aim of this study was to determine the incidence, biological characteristics and clinical features in association with molecular mutations, and the overall survival and outcome of patients with ET and PMF, presenting to our tertiary care centre from all the major provinces of Pakistan.

## Methods

### Study design

The study was prospective observational and conducted at National Institute of Blood Diseases & Bone Marrow Transplantation between 2012 and 2017. All procedures performed in studies involving human participants were in accordance with the ethical standards of the institutional research committee and with the 1964 Helsinki Declaration and its later amendments or comparable ethical standards. The study was approved by the ethics committee of NIBD and BMT (NIBD/RD-135/15–2012). Informed written consent was obtained from all patients before entering the data into the electronic database system.

### Diagnosis

ET and PMF were diagnosed according to World Health Organization (WHO) classification of Myeloid and Lymphoid Malignancies 2008 [15]. Complete blood count (CBC), bone marrow biopsy and molecular and cytogenetic analyses were recorded for each patient. A symptom-assessment form (SAF) was given to all patients at baseline and subsequent visits to avoid subjectivity in the assessment of the degree of constitutional symptoms and the effects on the quality of life of patients. Measurements for liver and spleen size were also recorded.

### Molecular and cytogenetic analysis

Cytogenetic analysis was performed using conventional G-banding techniques. The *JAK2* mutation was assessed using a polymerase chain reaction (PCR)-based amplification system [16]. Sanger sequencing was performed to detect the *MPL* W515L/K and *CALR* exon 9 mutations. Exon 10 of *MPL* was amplified using the following primers: F, 5′-TTCTGTACATGAGCATT- TCATCA-3′ and R, 5′-GACAGGCTGTGTGTGTGTACCTCT-3′. Exon 9 of *CALR* was amplified using the following primers: F, 5′-GAGGAGTTTGGCAA CGAGAC-3′ and R, 5′-AACCAAAATCCACCCCAAAT-3′.

### Risk stratification

Patients diagnosed with ET were categorized into high and low risk based on the presence or absence of thrombosis and age ≥ 60 years [17]. For patients with PMF, the DIPSS plus scoring system defined by the International Working Group (IWG) for MF was used to categorize patients into low, intermediate-1, intermediate-2 and high-risk groups [18].

### Assessment of response and disease progression

The response to treatment in ET was assessed according to revised-response criteria proposed by IWG-MRT [19]. All patients received 300 mg of aspirin. Platelet pheresis was offered to patients with platelet counts ≥1500 × 10^9^/L at baseline or those having thromboembolic manifestations regardless of platelet counts. Von Willebrand factor activity was checked in all patients with platelet counts of ≥1500 × 10^9^/L, to rule out acquired von Willebrand disease. High risk patients received cytoreductive therapy with hydroxyurea along with aspirin. Pegylated interferon or oral busulfan was offered to those intolerant or resistant to first-line treatment.

Response assessment in PMF was based on revised-response criteria proposed by IWG-MRT and ELN, including normalization of blood counts and age-adjusted normocellularity of bone marrow, resolution of constitutional symptoms and hepatosplenomegaly after a treatment of at least ≥12 ± weeks [20]. For symptomatic splenomegaly, hydroxyurea and for anaemia, erythropoiesis stimulating agents in combination with synthetic androgens were used. JAK2 inhibitor was offered to few patients, when it received FDA approval in 2014. The presence of circulating blasts and changes in the grade of bone marrow fibrosis from baseline was considered as sign of disease progression into post-ET MF or acute leukaemia.

### Statistical analysis

SPSS software (IBM SPSS Statistics, New York, USA, version 20.0) was used to calculate the frequency of qualitative variable i.e., gender and mean, median and standard deviation of quantitative variables such as age, haemoglobin, platelets and white blood cells. Continuous variables were analysed by using the Wilcoxon rank-sum test. Patient characteristics were compared using the Fisher’s exact test. Overall survival (OS) was defined as the time from diagnosis of ET or PMF to date of death (uncensored) or last contact (censored). Leukaemia-free survival (LFS) was calculated from the date of diagnosis to transformation into leukaemia. OS and LFS were plotted using Kaplan-Meier curves and compared by a log-rank test. *P* values< 0.05 were considered to indicate statistically significant differences.

## Results

### Frequencies of molecular and cytogenetic mutations

**Fig. 1:**
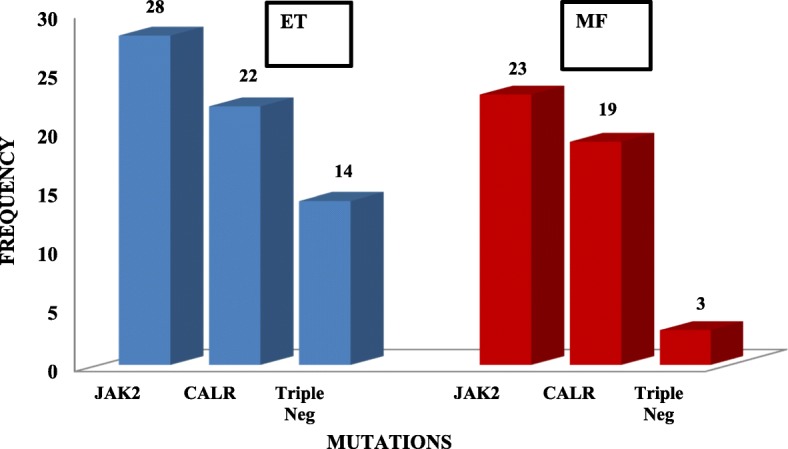
Distribution of JAK2 V617F, MPL, and CALR mutations in patients with essential thrombocythemia (ET) and primary myelofibrosis (PMF)

A total of 137 patients were analysed in this study, 75 patients were diagnosed with ET and 62 patients were diagnosed with PMF. *JAK2* positivity was seen in 51 cases (37.2), *CALR* in 41 cases (29.9%), and triple-negative in 17 (12.4%) cases. Of the 75 patients with ET, 28 (37.3%) harboured the *JAK2* mutation, and 22 (29.3%) harboured the *CALR* mutation. *MPL* mutation was not detected in any of the patients. Fourteen (18.7%) patients were triple-negative for all 3 mutations (Fig. 1). ET patients with *CALR* mutations accounted for 46.8% of patients who had non-mutated *JAK2.* Of the ET patients with *CALR* mutations, 13 (59.1%) had Type 1 mutation and 9 (40.9%) had Type 2 mutation.

Of the 62 patients with PMF, 23 (37.1%) harboured the *JAK2* mutation, 19 (30.6%) had *CALR* mutation and none of the patient harboured the *MPL* mutation. Three (4.8%) patients were negative for all 3 mutations (Fig. 1). PMF patients with *CALR* mutations accounted for 48.7% of the patients with non-mutated *JAK2*. Of those with mutated *CALR,* 52.6% had Type 1 *CALR* mutation while 47.7% had Type 2 *CALR* mutation. Homozygous *CALR* mutation was detected in one patient with the fibrotic phase of PMF, which was an exclusive finding, that has never been previously reported in MPN patients [21]. Six out of 7 patients with post-ET and post-PV MF harboured the *JAK2* mutation.

Cytogenetic analysis revealed an abnormal karyotype in 10 (7.2%) patients. The most common karyotypic abnormality detected was del20q in 5% of patients followed by trisomy + 8 and + 13 in small number of PMF patients.

### Clinico-haematologic features and genotype-phenotype correlation

Of patients with ET, 37 (49%) were male. According to 2013 ELN risk stratification, 52 (69.3%) were low risk patients and 23 (30.7%) were high risk patients. The median age of patients was 38 years (range: 19–56 years) and 71 years (range: 30–89 years) in the low and high-risk groups respectively. Splenomegaly was found in 35.7, 77.2 and 50% of *JAK2* positive, *CALR* positive and triple-negative patients respectively. Table 1 summarizes the clinical and haematological characteristics of the study patients based on molecular mutations. Among the 3 mutational groups, *JAK2* positive ET was associated with older age (58.5 ± 14.4 years) and large spleen size; *CALR* positive ET was associated with younger age (37 ± 10.4 years), higher platelet count (1191.9 ± 653.2 × 10^9^/L) and low haemoglobin levels (11.6 ± 2.2 g/dl) and triple-negative ET was associated with higher WBC count (19.1 ± 36.9 × 10^9^/L). Statistically significant differences were observed between the three groups for age (*p*-value: < 0.001) and spleen size (*p*-value: 0.007). Thromboembolic manifestations and constitutional symptoms were commonly observed in *JAK2* positive ET.

**Table 1:** Molecular and clinical characteristics of patients with essential thrombocythemia (ET)

Variables	*CALR* mutation (*n* = 22)	*JAK2 V617F* mutation (*n* = 28)	Triple Negative (*n* = 14)	*p*-value
**Males (%)**	15 (68.2)	12 (42.9)	6 (42.9)	0.395
**Age,** **Median (Range)**	35 (28–73)	57.5 (34.0–85.0)	35 (18–75)	0.001**
**Risk Group:**				
Low (%)	15	14	2	0.043*
High (%)	7	14	12	
**Haemoglobin (g/dL),** **Median (Range)**	10.2 (9.2–11.1)	12.3 (10.5–15.1)	12.8 (11.7–16.8)	0.641
**TLC ×10**^**9**^**/L,** **Median (Range)**	9.3 (5.11–16.7)	9.5 (4.7–147)	11.2 (2.3147)	0.061
**Platelet ×10**^**9**^**/L,** **Median (Range)**	1003.0 (462–2305)	928.5 (92–1883)	1064.5 (382–1841)	0.373
**Reticulin Fibrosis**				
**MF 0**	12 (54.5)	20 (71.4)	10 (71.4)	
**MF 1**	7 (318)	8 (28.6)	4 (28.6)	0.052
**MF 2**	3 (13.6)	0	0	
**Constitutional symptoms (%)**	13 (59.1)	13 (46.4)	4 (28.6)	
**Thromboembolic events (%)**	3 (13.6)	4 (14.3)	2(14.3)	0.033*

**highly significant, *significant

Of patients with PMF, 34 (54.8%) were male. The median age of patients was 52 years (range: 20–81 years). The study characteristics of PMF patients are shown in Table 2. According to DIPSS plus risk stratification, 4 (6.5%) were low risk, 7 (11.3%) were intermediate-1 risk, 27% (43.5%) were intermediate-2 risk and 7 (11.3%) were high risk patients. Splenomegaly and circulating blasts were found in 75.8 and 6.5% of patients at baseline respectively. *JAK2* positive PMF was associated with older age (53.0 ± 16.2 years) and intermediate-2 risk disease, whereas *CALR* positive PMF was also associated with intermediate-2 risk disease.

**Table 2:** Molecular and clinical characteristics of patients with primary myelofibrosis (PMF)

Variables	*CALR* mutation (*n* = 19)	*JAK2* mutation (*n* = 23)	Triple Negative (*n* = 3)	*p*-value
**Males (%)**	13 (68.4)	14 (60.9)	1 (33.3)	0.025*
**Age,** **Median (Range)**	45 (37–70)	53 (21–76)	43 (22–60)	0.138
**Haemoglobin (g/dL),** **Median (Range)**	10.5 (9.5–13.4)	9.8 (6.6–15)	7.9 (6.5–14.2)	0.45
**TLC ×10**^**9**^**/L,** **Median (Range)**	9.8 (5.6–63.2)	11.9 (1.8–22.1)	40.9 (15.1–25)	0.075
**Platelet × 10**^**9**^**/L,** **Median (Range)**	273 (122–1147)	382.5 (12–239)	131.5 (38–483)	0.358
**Circulating blasts (%),** **Median (Range)**	29	47.5 (44–51)	38	0.306
**Reticulin Fibrosis**				
**MF 0**	0	0	0	
**MF 1**	5 (26.3)	15 (65.2)	2 (66.7)	0.067
**MF 2**	14 (73.7)	8 (34.8)	1 (33.3)	
**Constitutional symptoms (%)**	12 (63.2)	14 (60.9)	3 (100)	0.421
**DIPSS score, (%):**				
Low	4 (21.1)	0	0	
Intermediate-1	2 (10.5)	5 (21.7)	0	0.259
Intermediate-2	9 (47.4)	15 (65.2)	3 (100)	
High	4 (21.1)	3 (13)	0	
**Leukemic transformation, (%)**	2 (10.5)	2 (8.7)	1 (33.3)	0.629

*significant

Triple-negative PMF was associated with the lowest haemoglobin (7.4 ± 1.2 g/dl) and platelet count (100.3 ± 62.0 × 10^9^/L) and the highest WBC count (40.6 ± 66.9 × 10^9^/L) among the 3 mutational groups.

### Response to therapy and leukaemic transformation

Complete response to first-line treatment was achieved in 25 (48.1%) and 12 (52.5%) of low and high-risk ET patients respectively. Platelet-pheresis was required in 3 (5.8%) and 7 (30.4%) of low and high-risk patients respectively at initial diagnosis. Five (6.6%) patients were found refractory/resistant to first-line treatment and responded to second line treatment (pegylated-interferon). Progression into myelofibrosis occurred in 3 (4%) of patients but none of the patients transformed into acute leukaemia. Among patients with PMF, 13 (54.2%) patients showed a response to treatment with conventional agents. Twenty-four (63.2%) patients treated with JAK2 inhibitor showed a significant reduction in spleen size and improvement in constitutional symptoms. Leukaemic transformation was observed in 5 (8.1%) of patients.

### Impact of molecular mutations on overall survival and prognosis

**Fig. 2:**
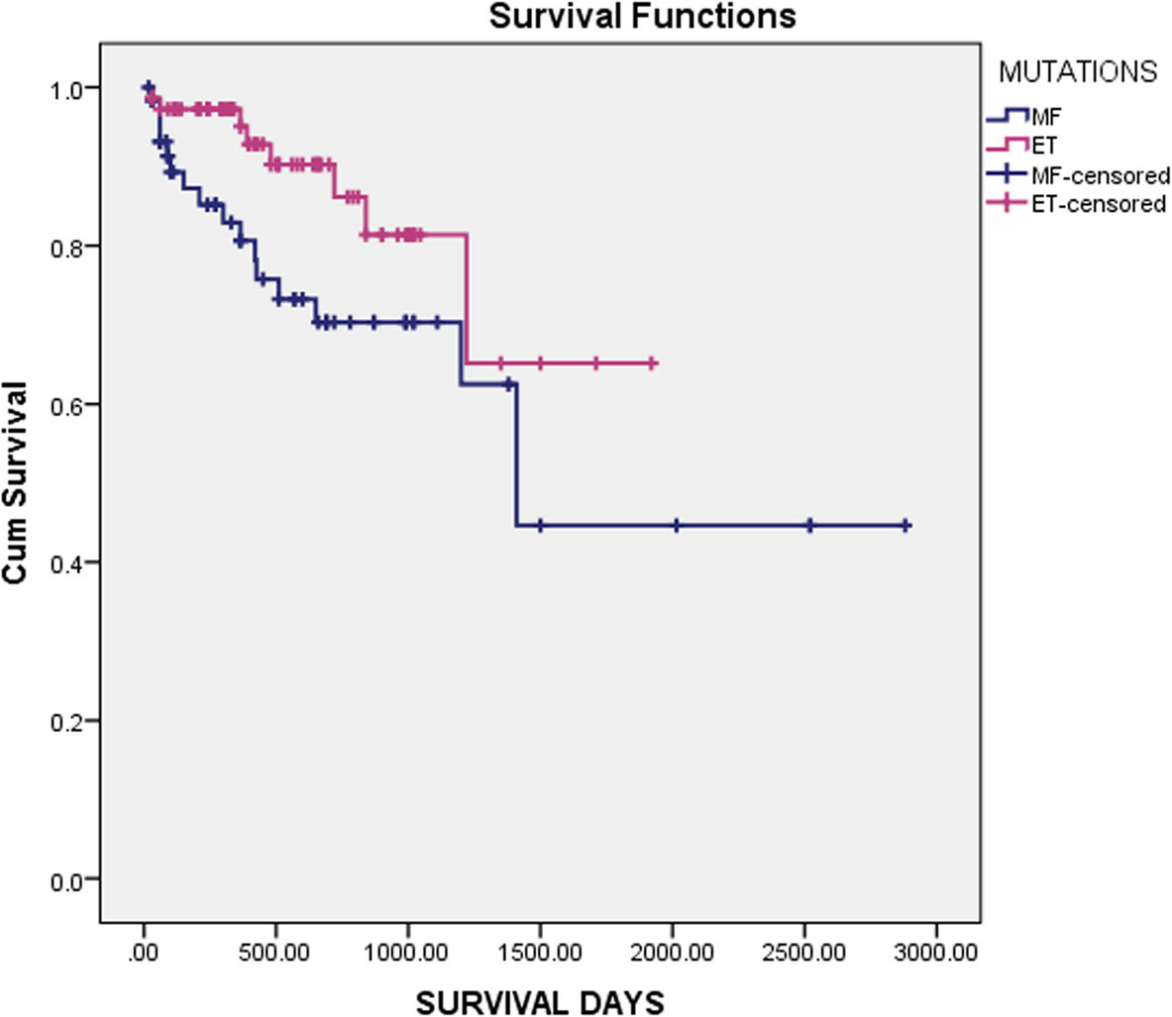
Overall Survival of the study participants

**Fig. 3:**
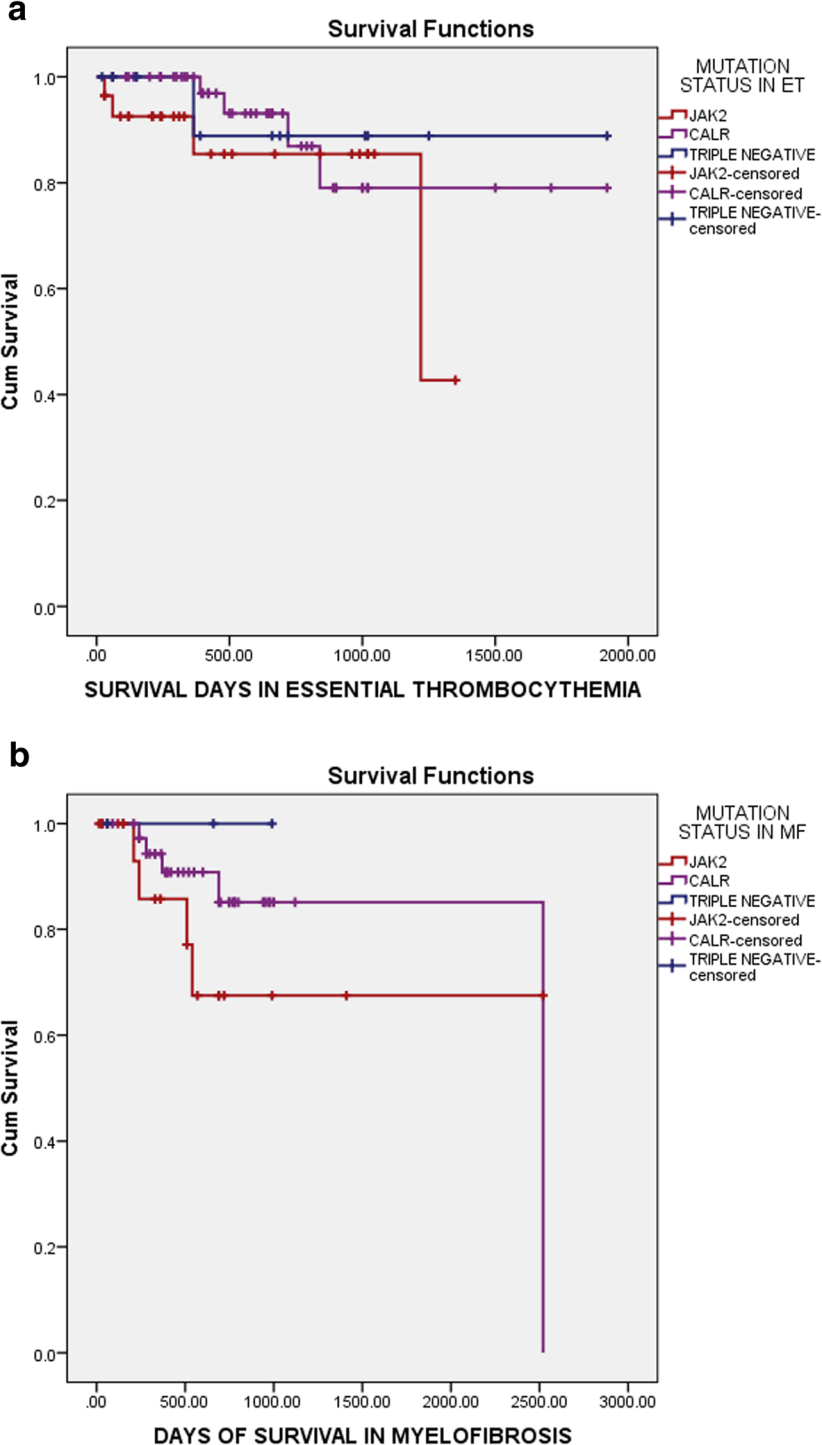
**a** Overall survival in essential thrombocythemia patients based on mutations. **b** Overall survival in primary myelofibrosis patients based on mutations

Overall survival for patients with ET and PMF was 92.5 and 86.0% respectively and leukaemia free survival for ET and MF was 100 and 91.6% respectively, at a median follow-up of 12 months (range:10–240 months) as shown in Fig. 2. None of the ET patients had leukaemic transformation while 8.1% of MF patients transformed and this transformation occurred more commonly in *JAK2* positive patients (*p* value = 0.377). Figure 3a shows that OS in ET was not affected by molecular mutational status whereas in PMF, OS was shortest in triple-negative group of patients (*p* value = 0.053) as shown in Fig. 3b. Among the other clinical parameters, univariate analysis found that an intermediate-2 DIPSS score was associated with significantly shorter OS (*p* = 0.234) and LFS (*p* = 0.032) than the intermediate-1 or high-risk group. *JAK2* mutation was associated with a higher risk of thromboembolic complications both in ET and PMF.

## Discussion

Driver mutations such as *JAK2, CALR* and *MPL* contribute to the heterogeneity in the phenotypic behaviour and outcome in patients with different subtypes of MPN [22–25]. This study presents the clinical and molecular profiles of ET and PMF patients from different regions of Pakistan to understand the differences in clinical presentation between the Pakistani population and other countries.

Data concerning the molecular mutations in MPN from Pakistan are scarce. Most of the literature related to MPN from South East Asian countries is from China and South Korea. The frequency of *JAK2* mutation reported in our study, is relatively lower than that reported in international studies; however the frequency of *CALR* and triple-negative MPN is consistent with those published in China and Korea. *JAK2V617F* was the first specific mutation identified in MPN pathogenesis, occurring with the highest frequency in polycythemia vera (81–99% of cases) followed by ET (41–72%) and MF (39–57%) and could be present as a heterozygous or homozygous mutation [26–29]. Bo Hyun Kim et al., reported the frequency of *JAK2* (51.2, 54%)*, CALR* (27.4, 22%) and triple-negative MPN (20.2, 20%), among 84 ET and 50 MF patients respectively, from Korea [30]. A similar study conducted by Li et al in 357 Chinese patients with PMF found that, 178 (50%) of patients carried *JAK2V617F*, 76 (21%) had a *CALR* mutation, 11 (3%) carried an *MPL* mutation, and 96 (27%) were triple-negative PMF [31]. Rumi et al., reported *JAK2 (*62%), *CALR (*24%), *MPL* (4%), and triple-negative *ET* (10%) among 745 European ET patients [32].

The incidence of *CALR* mutation in this study was in concordance with other studies from the Southeast Asian region but did not support the findings published in Western literature. Klampfl et al. initially reported a higher incidence of *CALR* mutations (67% in ET and 88% in PMF) in *JAK2* and *MPL* negative patients [6]. All the mutations identified occurred in exon 9 of the *CALR* gene. The ratio of Type 1 versus Type 2 *CALR* mutation in our study corresponds to that found in PMF and ET patients in Asian and European countries except for China, where this ratio is reversed i.e. Type 2 mutation is more prevalent in the Chinese population [31]. The prognostic value of Type 1 and Type 2 mutations has been discussed in various studies. Tefferi et al. showed that patients who carry the Type 1 CAL-R mutation had significantly longer survival than the patients with all other driver mutations [33].

Unexpectedly, none of the patients with ET and PMF in our study harboured the *MPL* mutation. *MPL* mutations may occur in as many as 8% of ET and MF patients, although the actual frequency of *MPL* mutations in MPN patients has not been as extensively studied as the prevalence of *JAK2* mutation [34]*.* Although very low frequency of *MPL* is reported in Korean population [30], the absence of *MPL* mutation in our population is a rare finding that needs confirmation in large scale studies.

The frequency of triple-negative MPN varies between 10 and 20% [35]. In our patients, triple-negativity was less commonly observed in PMF than ET. A European study reported 8.6% frequency of triple-negative PMF among 617 patients studied [36]. The ethnicity-based differences in the genetic profiles of the patients may be attributable to the incongruent findings observed in this study.

A small number of PMF patients in this study presented with cytogenetic abnormalities such as del20q and trisomy 8 at baseline. We did not find any statistically significant association of cytogenetic abnormalities with the molecular mutational profile of patients, and no clinical impact of these mutations could be observed on leukaemic transformation or overall survival of these patients. Approximately one third of patients with PMF present with cytogenetic abnormalities including del(20q), del(13q), trisomy 8 and 9, and abnormalities of chromosome 1 including duplication 1q. Patients with PMF that transform to acute leukaemia usually show complex karyotypes at transformation and a significantly decreased median survival [37, 38].

Overall, the clinical characteristics of our patients conformed to the results published in previous studies. In this study, *JAK2* mutation was associated with older age, high-risk disease and increased incidence of thrombosis or haemorrhage compared to *CALR* positive and triple-negative ET and PMF. The association of *JAK2* mutation with thromboembolism is well established in the literature. It is suggested that this mutation likely causes thrombosis through multiple mechanisms, including activation of platelets and granulocytes [39, 40]. More recently, the association of leucocytosis and *JAK2* mutation with thrombotic events has been confirmed in a retrospective study of 108 patients with ET [41]. Increased rate of vascular complications in ET have been associated with two variables, age and previous thrombotic history [42].

*CALR*-mutant ET and PMF have relatively indolent clinical course compared with the respective *JAK2*-mutant disorders [32]. In this study, *CALR* mutation was associated with higher platelet count, lower leukocyte count and low-risk disease. These findings correlate with previously published study [43]. Three large cohort studies reported that an increased baseline leukocyte count was an independent risk factor for both thrombosis and inferior survival in ET [44]. This might explain the lower incidence of thrombotic events and better overall survival associated with *CALR* mutations in ET. A recent evaluation of 709 consecutive Mayo Clinic patients with PMF, confirmed that survival was significantly longer with Type 1 *CALR*, compared to all other driver mutations, which were otherwise similar in their prognosis [33].

In our study, triple-negative ET and PMF were associated with lower haemoglobin levels and higher WBC counts. Triple- negative ET had a less severe disease course. Triple-negative PMF had more constitutional symptoms, high-risk disease and increased incidence of thrombo-embolic events at baseline. The risk of leukaemic transformation in triple-negative PMF was higher than the *JAK2* and *CALR*-mutated PMF in this study, leading to short OS in this group. These findings for triple-negative patients correlate with previously published studies from Asian and Western countries [45, 46]. Tefferi et al. have also highlighted the high-risk features of disease associated with triple-negative PMF [35].

Overall, the mutational status did not produce clinical impact on OS in ET, but in contrast, OS was found to be low in PMF patients who were triple-negative for all mutations as compared to *JAK2* and *CALR* mutated patients.

## Conclusion

This study shows a different spectrum of molecular mutations in ET and PMF patients in the Pakistani population compared to other Asian countries. Similarly, the risk of leukaemic transformation in ET and PMF is relatively lower in our population of patients. The factors responsible for these phenotypic and genotypic differences need to be analysed in large scale studies with longer follow up of patients.

The major limitations of this study include the relatively low numbers of patients in our cohort and lack of availability of next generation sequencing data for patients with triple-negative MPN.

## Data Availability

The datasets generated and analysed during the current study are not publicly available due to breach of confidentiality but are available from the corresponding author on reasonable request and after removing all the identifiable data.

## Abbreviations

CALR: Calreticulin
DIPSS: Dynamic International Prognostic Scoring System
ELN: European Leukaemianet
ET: Essential thrombocythemia
IWG - MRT: International Working Group-Myeloproliferative Neoplasms Research and Treatment
JAK2: Janus Kinase 2
LFS: Leukaemia-free Survival
MPL: Thrombopoietin receptor gene
MPNs: Myeloproliferative Neoplasms
OS: Overall survival
PMF: Primary myelofibrosis
SAF: Symptom-assessment form
WHO: World Health Organization
